# Association between triglyceride glucose–body mass index and acute kidney injury and renal replacement therapy in critically ill patients with sepsis: analysis of the MIMIC-IV database

**DOI:** 10.3389/fendo.2025.1561228

**Published:** 2025-07-21

**Authors:** Shijie Wang, Ruowen Li, Li Zhang, Tingbin Xie, Xinying Wang

**Affiliations:** Clinical Nutrition Service Center, Department of General Surgery, Nanjing jinling Hospital, Affiliated Hospital of Medical School, Nanjing University, Nanjing, China

**Keywords:** acute kidney injury, sepsis, triglyceride glucose-body mass index, predictor, insulin resistance

## Abstract

**Background:**

Previous studies have linked kidney damage to insulin resistance (IR), yet the association between triglyceride glucose–body mass (TyG–BMI) index, a reliable marker of IR, and acute kidney injury (AKI) remains unclear.

**Methods:**

Patient data were collected from the Medical Information Mart for Intensive Care IV (MIMIC-IV) database. AKI was set as the primary endpoint, and renal replacement therapy (RRT) was set as the secondary endpoint to represent the progression of AKI. TyG–BMI index and study endpoints were analyzed using Cox regression and restricted cubic spline (RCS) analyses.

**Results:**

A total of 1,117 patients with sepsis were enrolled, of whom 559 (50.0%) developed AKI. The result of Cox regression revealed that the TyG–BMI index was closely related to AKI (*P* = 0.032), and RCS analysis depicted a nonlinear correlation (*P* for nonlinear = 0.013). For RRT, similar results were observed. Compared with the simple severity of illness scores (SOFA, APSIII, SAPSII, and SIRS), when combined with the TyG–BMI index, their predictive ability for sepsis-related AKI significantly increased (AUCs: 0.745, 0.732, 0.708, and 0.566 vs. 0.756, 0.747, 0.728, and 0.661; all *P* < 0.05).

**Conclusions:**

For critically ill patients with sepsis, an elevated TyG–BMI index implies a possible increased risk of AKI. The TyG–BMI index has the potential to be a valuable predictor.

## Background

Sepsis, a life-threatening disease, is characterized by multi-organ damage induced by the dysfunction of the host’s immune response to infection (1). Annually, nearly 50 million cases are diagnosed globally, with sepsis-related deaths accounting for more than 50% of in-hospital deaths (2, 3). Despite advancements in medical technology, the mortality rates for sepsis have not significantly improved (4). Sepsis may impair renal function, with approximately 60% of patients experiencing acute kidney injury (AKI) (5, 6). Once it occurs, it increases sepsis mortality by three to five times, leading to worse clinical outcomes (7). Therefore, the early detection of patients with a tendency to develop AKI and timely intervention are crucial to improve the prognosis.

Sepsis is often accompanied by insulin resistance (IR), which may be caused by systemic inflammation (8). Additionally, IR can inhibit the autophagic activity of podocytes, leading to kidney injury, and is positively correlated with kidney injury molecule-1 (9, 10). The triglyceride–glucose (TyG) index, an innovative marker, has been considered a convenient replacement indicator for IR (11). More importantly, the degree of IR in the body is more accurately reflected when used in conjunction with body mass index (BMI) (12). The findings above seem to suggest that TyG–BMI index may predict the occurrence of AKI, which would help to identify high-risk patients and thus enable early intervention. For certain diseases, such as hypertension, myocardial infarction, and chronic kidney disease, a strong association exists between their incidence and TyG–BMI index (13–15). However, it remains unclear whether this correlation exists in individuals with sepsis-associated AKI.

Consequently, the current study hypothesizes an association between TyG–BMI index and sepsis-associated AKI and intends to explore the issue utilizing this large cohort, with a view to guiding clinical practice.

## Methods

### Study population

Clinical data were retrospectively extracted from the MIMIC-IV database. One author (WSJ) successfully passed all of the required examinations for accessing the database and obtained approval to use the dataset (certification number: 56051808). The review committee of Massachusetts Institute of Technology and Beth Israel Deaconess Medical Center approved the database for medical health-related research without requiring informed consent.

**Figure 1:**
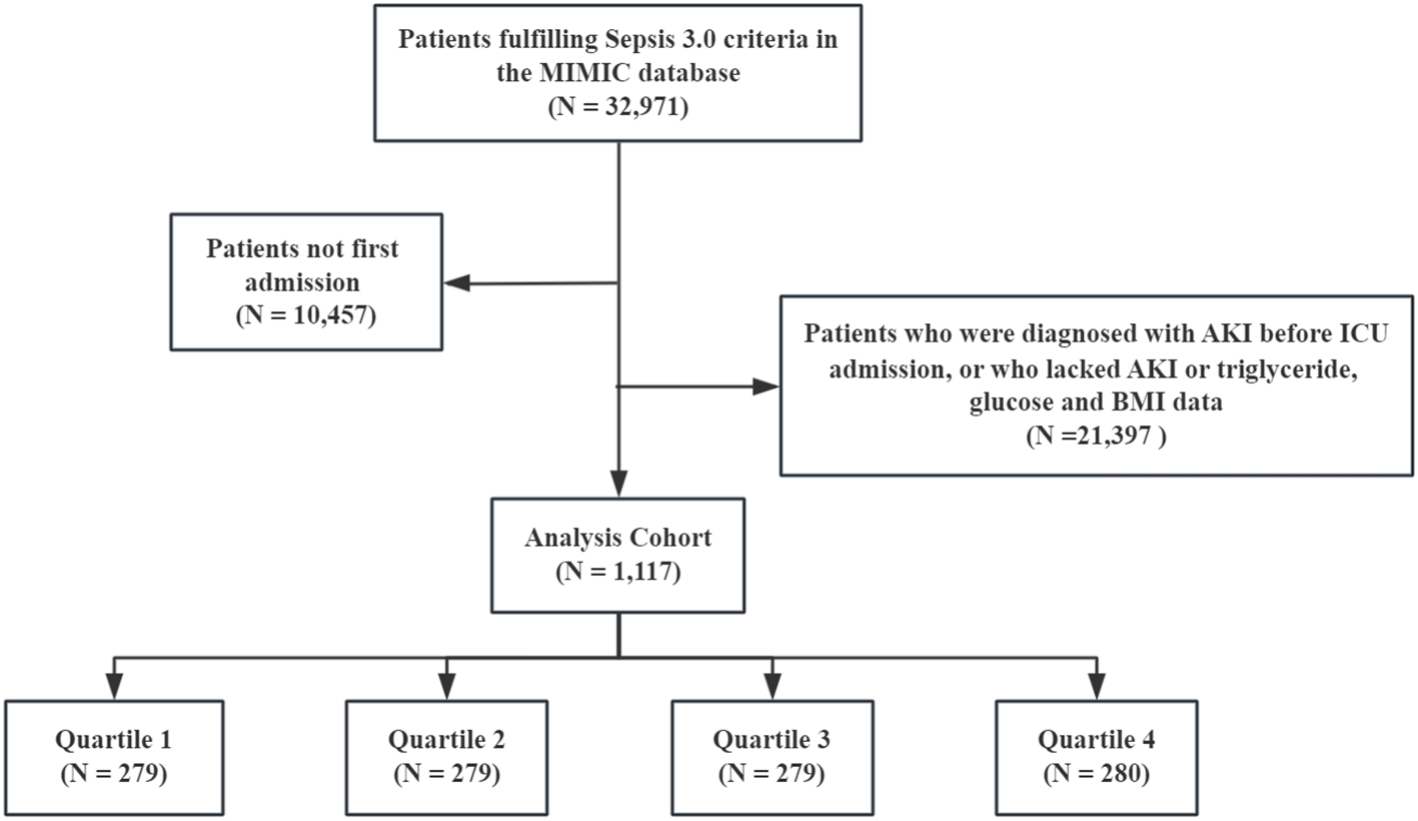
Flow of the included patients.

All patients with sepsis met the Sepsis 3.0 criteria, defined as the presence of infection and sequential organ failure assessment (SOFA) score ≥2 (16). The Kidney Disease: Improving Global Outcomes (KDIGO) guideline was used to confirm the presence of AKI (17). The exclusion criteria were as follows: (1) age <18 years, (2) only the first data were extracted if multiple ICU admissions for sepsis existed, (3) missing fasting blood glucose (FBG), triglyceride, and BMI data within 24 h of ICU admission, (4) diagnosed with AKI prior to ICU admission, (5) and missing AKI data within 48 h. Finally, 1,117 patients with sepsis were enrolled, and the cohort was divided according to the TyG–BMI quartile (**Figure 1**).

### Data collection

Data on the basic characteristics of the patients were extracted using PostgresSQL and Navicat Premium software and merged with Stata software. Detailed clinical data included demographics (age, sex, race, height, weight, and BMI), laboratory test results (international normalized ratio [INR], blood urea nitrogen [BUN], low-density lipoprotein [LDL], sodium, chloride, aspartate aminotransferase [AST], albumin, red blood cell [RBC], high-density lipoprotein [HDL], neutrophils, hemoglobin, platelets, hematocrit, total bilirubin, prothrombin time [PT], C-reactive protein [CRP], serum creatinine [SCr], alanine aminotransferase [ALT], white blood cells [WBC], alkaline phosphatase [ALP], calcium, lymphocytes, anion gap, activated partial thromboplastin time [APTT], blood glucose, triglycerides, potassium, and total cholesterol [TC]), medication (statin, insulin, and metformin), vital signs, and severity of illness scores. Since the MIMIC database does not explicitly specify which values represent FBG, all values are simply labeled as “blood glucose”. Therefore, we inferred based on patients’ medication use to exclude interference from insulin, glucose injection, and enteral nutrition on blood glucose values as much as possible. Data on the blood collection time for glucose tests on the first day of ICU admission as well as the start times of insulin, glucose injections, and enteral nutrition were extracted. If the blood collection occurred after the start of any of these interventions, the corresponding glucose value was considered interfered and excluded. Specific procedures and codes are provided in the supplementary methods. The International Classification of Diseases (9th and 10th) was used to identify comorbidities, including chronic kidney disease (CKD), cancer, diabetes, hypertension, arterial fibrillation (AF), and heart failure (HF). Within 24 h of ICU admission, all test indicators and scores were collected, and the SCr level and urine output were continuously monitored throughout the hospital stay. Information on medication use in the 24 h prior to ICU admission was collected. The timings of initial AKI and renal replacement therapy (RRT) were determined. Follow-up continued from the date of admission to all study endpoints.

TyG–BMI index formula: ln [triglyceride (mg/dL) × FBG (mg/dL)/2] × BMI (18). For the variables included in this study, multiple interpolation (multiple imputation by chained equations) was used to fill in those with missing values <20%, while those with missing values >20% were deleted (12). Lymphocytes, neutrophils, albumin, HDL, LDL, CRP, and TC contained more than 20% missing value.

### Endpoints of interest

AKI was set as the primary endpoint. KDIGO guidelines were utilized: SCr was 1.5-fold higher than baseline within 7 days or elevation of SCr by 0.3 mg/dL in 48 h or urine output less than 0.5 mL/kg per hour for at least 6 h. The reference baseline of SCr was determined as the lowest recorded value within 7 days prior to ICU admission (276 patients), and if this information was not available, the SCr first measured at admission to ICU was used (841 patients). RRT, representing disease progression to AKI, was used as a secondary endpoint. Meanwhile, ICU, in-hospital, 28-day, and 1-year mortality were also specified as secondary endpoints.

### Statistical analysis

The proportional hazards assumption was verified using Schoenfeld residual plots, and no violation was detected (**Supplementary Figure S1**). The occurrence of primary and secondary endpoints was depicted by the Kaplan–Meier curve. By utilizing Cox regression analysis, the study excluded confounders to identify independent association (survival package, version 3.5–5 and survminer package, version 0.4.9). The Fine–Gray model was constructed to analyze the competitive risk in order to evaluate the stability of the results (cmprsk package). To evaluate the possible influence of unmeasured confounding on the observed hazard ratios, E-values were analyzed. To avoid multicollinearity, variables were excluded when the variance inflation factor was greater than 5 (car package). To depict the dose–response effects, restricted cubic spline (RCS) analysis was conducted (ggrcs package, version 0.4.0). Furthermore, subgroup analyses of hypertension, HF, CHD, CKD, AF, diabetes, age, sex, and BMI were conducted (jstable package, version 1.1.7). The interactions were assessed with likelihood ratio tests.

The area under the curve (AUC) was used to reflect the predictive power of existing severity of illness scores for AKI when incorporating the TyG–BMI index (timeROC package). Integrated discrimination improvement (IDI) was computed by subtracting the difference in the probability of positivity predicted by the difference between the different models for the disease group from the difference in the probability of positivity predicted by the old and new models for the non-disease group (19). Reclassified by event occurrence, the net reclassification improvement (NRI) performed a net magnitude synthesis and quantified the degree of improvement. These two indexes allow the risk reclassification of the model (survIDINRI package, version 1.1–2). The analysis and visualization were conducted using R (version 4.1.3) and SPSS (version 27.0). Statistical significance in the current study was defined as *P <*0.05.

## Results

### Patient characteristics

A total of 1,117 patients with sepsis were enrolled, of whom 559 (50.0%) developed AKI and 201 (18.0%) received RRT. Meanwhile, 204 (18.5%) ICU deaths and 250 (22.4%) in-hospital deaths occurred.

According to the TyG–BMI index, the overall patients were grouped by quartiles [quartile (Q) 1: <244.37; Q2: 244.37–291.05; Q3: 291.06–355.40; Q4: >355.40]. Patients in the Q4 group had higher BMI, heart rate, and severity of illness scores but were younger. The prevalence of diabetes was higher, and that of CHD was lower in this group. With regard to laboratory indicators, the Q4 group showed higher WBC, anion gap, total bilirubin, ALT, AST, BUN, SCr, FBG, triglycerides, and potassium ions but lower platelets, chloride, and calcium levels. With increasing TyG–BMI index, the incidence of AKI and RRT in the four groups gradually increased (AKI: 36.2% vs. 43.7% vs. 53.4% vs. 66.8%, *P* < 0.001; RRT: 5.7% vs. 11.8% vs. 24.7% vs. 29.7%, *P* < 0.001). However, no statistical differences were observed in the in-hospital, ICU, 28-day, and 1-year mortality among the groups (*P* = 0.552, *P* = 0.323, *P* = 0.827, and *P* = 0.697, respectively) (**Table 1**).

**Table 1:** Baseline characteristics according to TyG–BMI index quartiles.

Variables	Overall (*N* = 1,117)	Q1 (*N* = 279)	Q2 (*N* = 279)	Q3 (*N* = 279)	Q4 (*N* = 280)	*P*-value
Demographics						
Age (years)	63.52 (51.72, 74.82)	66.16 (51.35, 79.72)	66.53 (53.44, 80.29)	60.96 (51.75, 70.31)	61.42 (48.61, 69.38)	<0.001
Sex (male)	680 (60.9%)	155 (55.6%)	170 (60.9%)	194 (69.5%)	161 (57.5%)	0.004
Race (white)	849 (76.0%)	210 (75.3%)	217 (77.8%)	213 (76.3%)	209 (74.6%)	0.315
Height (cm)	170 (163, 178)	170 (160, 175)	170 (163, 178)	173 (165, 178)	170 (163, 178)	<0.001
Weight (kg)	85.1 (70.0, 101.8)	63.6 (55.2, 72.0)	77.3 (70.0, 87.5)	95.0 (84.4, 102.8)	112.9 (98.5, 129.9)	<0.001
BMI (kg/m^2^)	28.96 (25.05, 34.04)	22.42 (20.52, 24.30)	27.16 (25.72, 28.62)	31.23 (29.38, 33.33)	38.66 (35.31, 44.11)	<0.001
Infection site						
Lung	353 (31.6%)	82 (29.4%)	88 (31.5%)	86 (30.8%)	97 (34.6%)	0.688
Abdomen	175 (15.7%)	39 (14.0%)	46 (16.5%)	47 (16.9%)	43 (15.4%)	
Urinary system	261 (23.4%)	62 (22.2%)	71 (25.5%)	66 (23.7%)	62 (22.1%)	
Other	328 (29.4%)	96 (34.4%)	74 (26.5%)	80 (28.7%)	78 (27.9%)	
Infection type						
Gram-positive	388 (34.7%)	97 (34.8%)	87 (31.2%)	98 (35.1%)	106 (37.9%)	0.572
Gram-negative	343 (30.7%)	87 (31.2%)	85 (30.5%)	82 (29.4%)	89 (31.8%)	
Other	386 (34.6%)	95 (34.1%)	107 (38.4%)	99 (35.5%)	85 (30.4%)	
Laboratory tests						
Hemoglobin (g/dL)	10.6 (8.6, 12.3)	10.6 (8.5, 12.2)	10.6 (8.8, 12.3)	10.4 (8.5, 12.5)	10.6 (8.7, 12.4)	0.855
Platelets (K/uL)	204 (146, 280)	217 (154, 296)	202 (141, 271)	199 (141, 274)	198 (147, 267)	0.048
Hematocrit (%)	31.9 (26.3, 37.1)	31.9 (26.0, 36.6)	32.0 (26.5, 36.9)	31.7 (25.7, 37.8)	31.9 (26.5, 37.4)	0.553
WBC (K/uL)	14.4 (10.3, 19.0)	13.7 (10.0, 17.8)	13.5 (9.8, 17.6)	15.1 (10.3, 19.8)	15.7 (11.3, 21.2)	<0.001
RBC (K/µL)	3.81 (3.24, 4.39)	3.70 (3.14, 4.35)	3.81 (3.27, 4.32)	3.84 (3.17, 4.39)	3.96 (3.31, 4.51)	0.060
Anion gap (mEq/L)	17 (15, 20)	16 (14, 19)	17 (15, 20)	17 (15, 22)	18 (15, 21)	<0.001
Total bilirubin (mg/dL)	0.8 (0.5, 1.6)	0.8 (0.5, 1.1)	0.8 (0.6, 1.5)	0.8 (0.5, 1.8)	0.9 (0.5, 2.1)	<0.001
INR	1.3 (1.2, 1.6)	1.3 (1.1, 1.6)	1.3 (1.1, 1.6)	1.3 (1.2, 1.8)	1.3 (1.2, 1.6)	0.072
Prothrombin time	14.4 (12.8, 17.8)	14.3 (12.6, 17.5)	14.4 (12.7, 17.4)	14.7 (13.0, 19.8)	14.4 (12.8, 17.4)	0.101
APTT	33.1 (28.2, 50.6)	32.5 (28.6, 50.8)	31.5 (27.7, 46.5)	34.7 (28.5, 57.6)	33.7 (28.1, 48.1)	0.039
ALT (U/L)	41 (21, 88)	36 (16, 78)	39 (21, 85)	48 (23, 131)	42 (25, 94)	0.003
ALP (U/L)	82 (66, 109)	80 (64, 108)	80 (63, 103)	84 (66, 111)	86 (67, 114)	0.096
AST (U/L)	65 (32, 117)	54 (28, 127)	64 (28, 179)	70 (36, 272)	71 (37, 186)	0.005
BUN (mg/dL)	24 (16, 40)	20 (15, 34)	21 (15, 34)	25 (18, 45)	29 (19, 48)	<0.001
Serum creatinine (mg/dL)	1.2 (0.9, 2.1)	1.1 (0.7, 1.5)	1.2 (0.8, 1.7)	1.3 (1.0, 2.4)	1.6 (1.0, 2.7)	<0.001
Glucose (mg/dL)	159 (129, 226)	140 (116, 183)	150 (126, 205)	178 (135, 258)	191 (143, 254)	<0.001
Triglycerides (mg/dl)	123 (85, 207)	89 (65, 123)	113 (82, 164)	137 (97, 228)	205 (119, 382)	<0.001
Sodium (mEq/L)	141 (138, 144)	141 (138, 144)	141 (138, 144)	141 (138, 144)	140 (137, 144)	0.143
Chloride (mEq/L)	106 (102, 110)	107 (103, 111)	106 (103, 110)	106 (102, 110)	105 (101, 109)	0.021
Potassium (mEq/L)	4.5 (4.1, 5.0)	4.3 (4.0, 4.8)	4.4 (4.1, 4.8)	4.5 (4.1, 5.3)	4.7 (4.1, 5.5)	<0.001
Calcium (mg/dL)	8.0 (7.3, 8.5)	8.0 (7.5, 8.4)	8.1 (7.4, 8.7)	7.9 (7.2, 8.4)	7.8 (7.1, 8.4)	0.005
Vital signs						
SBP (mmHg)	118 (107,133)	118 (107,134)	120 (108, 134)	117 (107,133)	116 (105, 130)	0.308
DBP (mmHg)	63 (56, 71)	63 (56, 71)	64 (57, 72)	64 (51, 71)	62 (55, 70)	0.224
MBP (mmHg)	79 (72, 87)	78 (67, 87)	88 (80, 99)	79 (72, 87)	77 (71, 86)	0.074
Heart rate (beats/min)	86 (75, 99)	82 (70, 95)	84 (74, 96)	85 (75, 98)	92 (78, 106)	<0.001
Medication						
Statin	138 (12.4%)	28 (10.0%)	31 (11.1%)	37 (13.3%)	42 (15.0%)	0.283
Insulin	176 (15.8%)	36 (12.9%)	46 (16.5%)	46 (16.5%)	48 (17.1%)	0.505
Metformin	38 (3.4%)	7 (2.5%)	6 (2.2%)	16 (5.73%)	9 (3.2%)	0.084
Severity of illness scores						
SOFA score	6 (4, 10)	5 (3, 8)	5 (3, 9)	7 (4, 11)	8 (5, 11)	<0.001
SIRS score	3 (2, 4)	3 (2, 3)	3 (2, 3)	3 (2, 4)	3 (3, 4)	<0.001
APSIII	50 (37, 69)	44 (35, 60)	45 (33, 59)	47 (34, 62)	51 (36, 75)	<0.001
SAPSII	39 (30, 50)	37 (29, 47)	38 (29, 48)	40 (30, 53)	42 (30, 53)	<0.001
Comorbidities						
Hypertension	479 (42.9%)	102 (36.6%)	129 (46.2%)	121 (43.4%)	127 (45.4%)	0.087
Heart failure	207 (18.5%)	46 (16.5%)	50 (17.9%)	62 (22.2%)	49 (17.5%)	0.314
CHD	261 (23.4%)	72 (25.8%)	76 (27.2%)	72 (25.8%)	41 (14.6%)	0.001
Arterial fibrillation	150 (13.4%)	37 (13.3%)	44 (15.8%)	32 (11.5%)	37 (13.2)	0.521
CKD	151 (13.5%)	34 (12.2%)	34 (12.2%)	39 (14.0%)	44 (15.7%)	0.562
Diabetes	318 (28.5%)	49 (17.6%)	65 (23.3%)	76 (27.2%)	128 (45.7%)	<0.001
Cancer	112 (10.0%)	30 (10.8%)	32 (11.5%)	26 (9.3%)	24 (8.6%)	0.655
Outcomes						
AKI	559 (50.0%)	101 (36.2%)	122 (43.7%)	149 (53.4%)	187 (66.8%)	<0.001
RRT	201 (18.0%)	16 (5.7%)	33 (11.8%)	69 (24.7%)	83 (29.7%)	<0.001
ICU mortality	204 (18.5%)	43 (15.4%)	48 (17.2%)	59 (21.1%)	54 (19.4%)	0.323
In-hospital mortality	250 (22.4%)	56 (20.1%)	63 (22.6%)	70 (25.1%)	61 (21.8%)	0.552
28-day mortality	285 (25.5%)	75 (26.9%)	71 (25.5%)	73 (26.2%)	66 (23.7%)	0.827
1-year mortality	430 (38.5%)	115 (41.2%)	108 (38.7%)	102 (36.6%)	105 (37.5%)	0.697

TyG–BMI index: Q1 (<244.37), Q2 (244.37–291.05), Q3 (291.06–355.40), and Q4 (>355.40).

BMI, body mass index; WBC, white blood cell; RBC, red blood cell; INR, international normalized ratio; APTT, activated partial thromboplastin time; ALT, alanine aminotransferase; ALP, alkaline phosphatase; AST, aspartate aminotransferase; BUN, blood urea nitrogen; SBP, systolic blood pressure; DBP, diastolic blood pressure; MBP, mean blood pressure; SOFA, sequential organ failure assessment; SIRS, systemic inflammatory response syndrome; APSIII, acute physiology score III; SAPSII, simplified acute physiological score II; CHD, coronary heart disease; CKD, chronic kidney disease; AKI, acute kidney injury; RRT, renal replacement therapy; ICU, intensive care unit.

Further grouping was determined by the presence of AKI (**Table 2**). The prevalence of HF, CKD, cancer, and diabetes was higher in the AKI group but with a lower prevalence of hypertension. Meanwhile, the BMI and severity of illness scores were also higher. For laboratory indicators, the AKI group had significantly higher levels of WBC, anion gap, total bilirubin, INR, PT, APTT, ALT, ALP, AST, BUN, SCr, FBG, triglycerides, and potassium. More importantly, the AKI patients showed a higher TyG–BMI index (*P* < 0.001).

**Table 2:** Baseline characteristics of the AKI and non-AKI groups.

Variables	Overall (*N* = 1,117)	Non-AKI (*N* = 558)	AKI (*N* = 559)	*P*-value
Demographics				
Age (years)	63.52 (51.72, 74.82)	62.58 (50.47, 76.73)	64.17 (52.93,74.08)	0.422
Sex (male)	680 (60.9%)	308 (55.2%)	372 (66.5%)	<0.001
Race(white)	849 (76.0%)	425 (76.2%)	424 (75.8%)	0.387
Height (cm)	170 (163, 178)	170 (163, 178)	173 (163, 178)	<0.001
Weight (kg)	85.1 (70.0, 101.8)	80.2 (67.9, 96.3)	90.0 (72.6, 106.1)	<0.001
BMI (kg/m2)	28.96 (25.05, 34.04)	27.95 (24.19, 32.67)	30.16 (25.78, 35.74)	<0.001
Infection site				0.023
Lung	353 (31.6%)	169 (30.3%)	184 (32.9%)	
Abdomen	175 (15.7%)	72 (12.9%)	103 (18.4%)	
Urinary system	261 (23.4%)	141 (25.3%)	120 (21.5%)	
Other	328 (29.4%)	176 (31.5%)	152 (27.2%)	
Infection type				0.113
Gram-positive	388 (34.7%)	208 (37.3%)	180 (32.2%)	
Gram-negative	343 (30.7%)	172 (30.8%)	171 (30.6%)	
Other	386 (34.6%)	178 (31.9%)	208 (37.2%)	
Laboratory tests				
Hemoglobin (g/dL)	10.6 (8.6, 12.3)	11.1 (9.5, 12.8)	9.9 (8.0, 11.8)	<0.001
Platelets (K/uL)	204 (146, 280)	221 (165, 299)	185 (129, 259)	<0.001
Hematocrit (%)	31.9 (26.3, 37.1)	33.5 (28.8, 38.3)	29.8 (24.5, 35.5)	<0.001
WBC (K/uL)	14.4 (10.3, 19.0)	13.8 (10.1, 17.6)	14.9 (10.7, 21.1)	<0.001
RBC (K/µL)	3.81 (3.24, 4.39)	3.97 (3.38, 4.46)	3.71 (3.03, 4.3)	<0.001
Anion gap (mEq/L)	17 (15, 20)	16 (14, 18)	19 (16, 24)	<0.001
Total bilirubin (mg/dL)	0.8 (0.5, 1.6)	0.8 (0.5, 1.0)	1.0 (0.6, 2.7)	<0.001
INR	1.3 (1.2, 1.6)	1.2 (1.1, 1.4)	1.4 (1.2, 1.9)	<0.001
PT	14.4 (12.8, 17.8)	13.7 (12.5, 15.8)	15.4 (13.3, 21.1)	<0.001
APTT	33.1 (28.2, 50.6)	31.3 (27.2, 39.8)	36.3 (30.2, 58.5)	<0.001
ALT (U/L)	41 (21, 88)	36 (20, 68)	50 (23, 165)	<0.001
ALP (U/L)	82 (66, 109)	80 (65, 97)	86 (66, 128)	0.001
AST (U/L)	65 (32, 117)	50 (27, 108)	98 (41, 375)	<0.001
BUN (mg/dL)	24 (16, 40)	17 (13, 23)	36 (24, 58)	<0.001
SCr (mg/dL)	1.2 (0.9, 2.1)	0.9 (0.7, 1.1)	2.0 (1.4, 3.1)	<0.001
Glucose (mg/dL)	159 (129, 226)	147 (122, 197)	178 (137, 250)	<0.001
Triglycerides (mg/dl)	123 (85, 207)	109 (75, 172)	141 (98, 252)	<0.001
TyG–BMI index	290.88 (244.64, 355.93)	274.58 (233.96, 330.39)	309.96 (256.67, 384.85)	<0.001
Sodium (mEq/L)	141 (138, 144)	141 (139, 144)	141 (137, 144)	0.111
Chloride (mEq/L)	106 (102, 110)	106 (103, 110)	106 (101, 111)	0.067
Potassium (mEq/L)	4.5(4.1, 5.0)	4.3 (4.0, 4.7)	4.7 (4.2, 5.4)	<0.001
Calcium (mg/dL)	8.0 (7.3, 8.5)	8.1 (7.6, 8.6)	7.7 (6.9, 8.2)	<0.001
Vital signs				
SBP (mmHg)	118 (107, 133)	121 (109, 136)	114 (104, 127)	<0.001
DBP (mmHg)	63 (56, 71)	65 (58, 73)	62 (55, 69)	<0.001
MBP (mmHg)	79 (72, 87)	80 (68, 88)	76 (70, 84)	<0.001
HR (beats/min)	86 (75, 99)	83 (72, 95)	89 (78, 103)	<0.001
Severity of illness scores				
SOFA score	6 (4, 10)	5 (3, 7)	9 (6, 12)	<0.001
SIRS score	3 (2, 4)	3 (2, 3)	3 (3, 4)	<0.001
APSIII	50 (37, 69)	41 (31, 51)	64 (48, 84)	<0.001
SAPSII	39 (30, 50)	33 (26, 42)	47 (37, 57)	<0.001
Medication				
Statin	138 (12.4%)	60 (10.8%)	78 (13.9%)	0.104
Insulin	176 (15.8%)	85 (15.2%)	91 (16.3%)	0.631
Metformin	38 (3.3%)	17 (3.0%)	21 (3.8%)	0.513
Comorbidities				
Hypertension	479 (42.9%)	263 (47.1%)	216 (38.6%)	0.004
Heart failure	207 (18.5%)	81 (14.5%)	126 (22.5%)	0.001
CHD	261 (23.4%)	128 (22.9%)	133 (23.8%)	0.736
Arterial fibrillation	150 (13.4%)	79 (14.2%)	71 (12.7%)	0.475
CKD	151 (13.5%)	37 (6.6%)	114 (20.4%)	<0.001
Diabetes	318 (28.5%)	135 (24.1%)	183 (32.7%)	0.002
Cancer	112 (10.0%)	45 (8.1%)	67 (12.0%)	0.002

BMI, body mass index; WBC, white blood cell; RBC, red blood cell; INR, international normalized ratio; PT, prothrombin time; APTT, activated partial thromboplastin time; ALT, alanine aminotransferase; ALP, alkaline phosphatase; AST, aspartate aminotransferase; BUN, blood urea nitrogen; SCr, serum creatinine; SBP, systolic blood pressure; DBP, diastolic blood pressure; MBP, mean blood pressure; HR, heart rate; SOFA, sequential organ failure assessment; SIRS, systemic inflammatory response syndrome; APSIII, acute physiology score III; SAPSII, simplified acute physiological score II; CHD, coronary heart disease; CKD, chronic kidney disease.

### Primary and secondary endpoints

**Figure 2:**
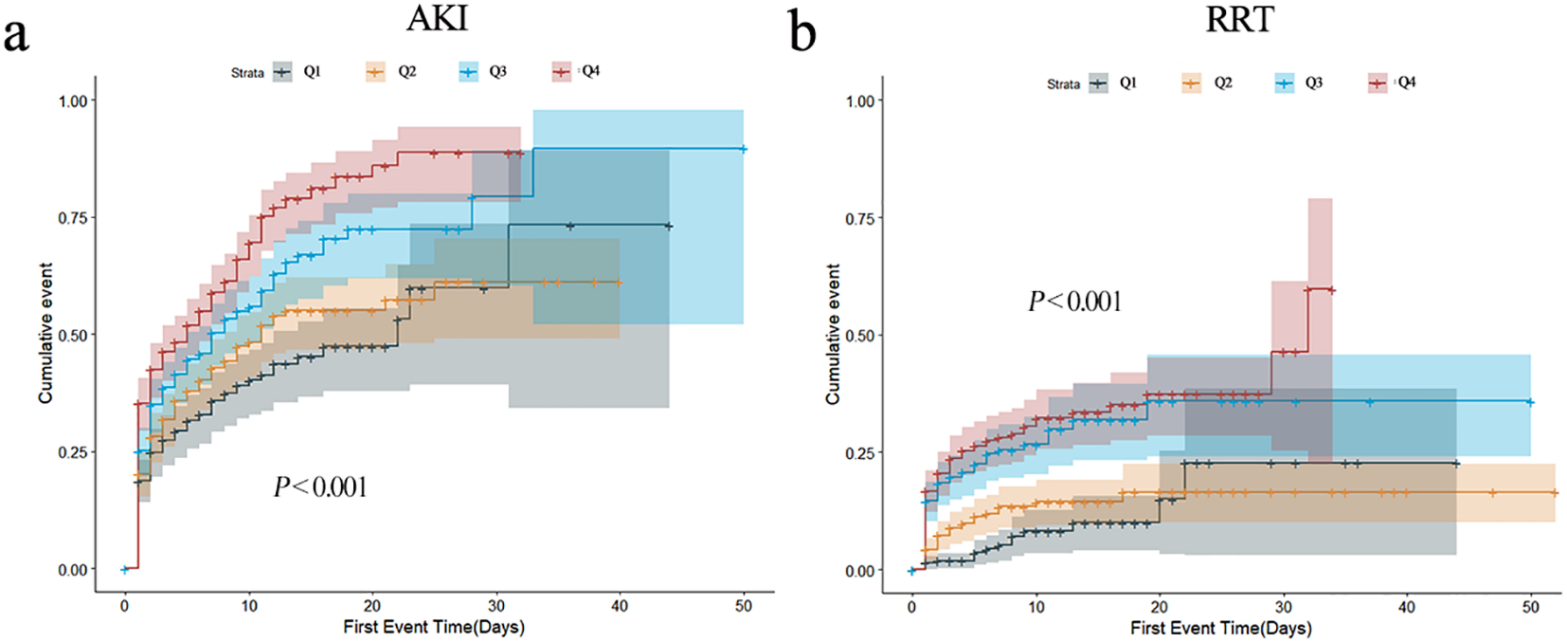
Cumulative event incidence curves for incidence of AKI **(a)** and requiring RRT **(b)**.

In order to describe the occurrence of study endpoints, Kaplan–Meier method was employed. For AKI, significant differences were observed; the Q4 group had the highest incidence of AKI (*P* < 0.001) (**Figure 2a**). As for RRT, similar results were observed (*P* < 0.001) (**Figure 2b**). The cumulative incident curves of AKI and RRT were plotted using the CIF method, and Gray’s test was conducted, showing similar results to the data above (*P* < 0.001) (**Supplementary Figure S2**). Nevertheless, no statistical differences existed among the four groups for other secondary endpoints (all *P* > 0.05) (**Supplementary Figure S3**).

**Figure 3:**
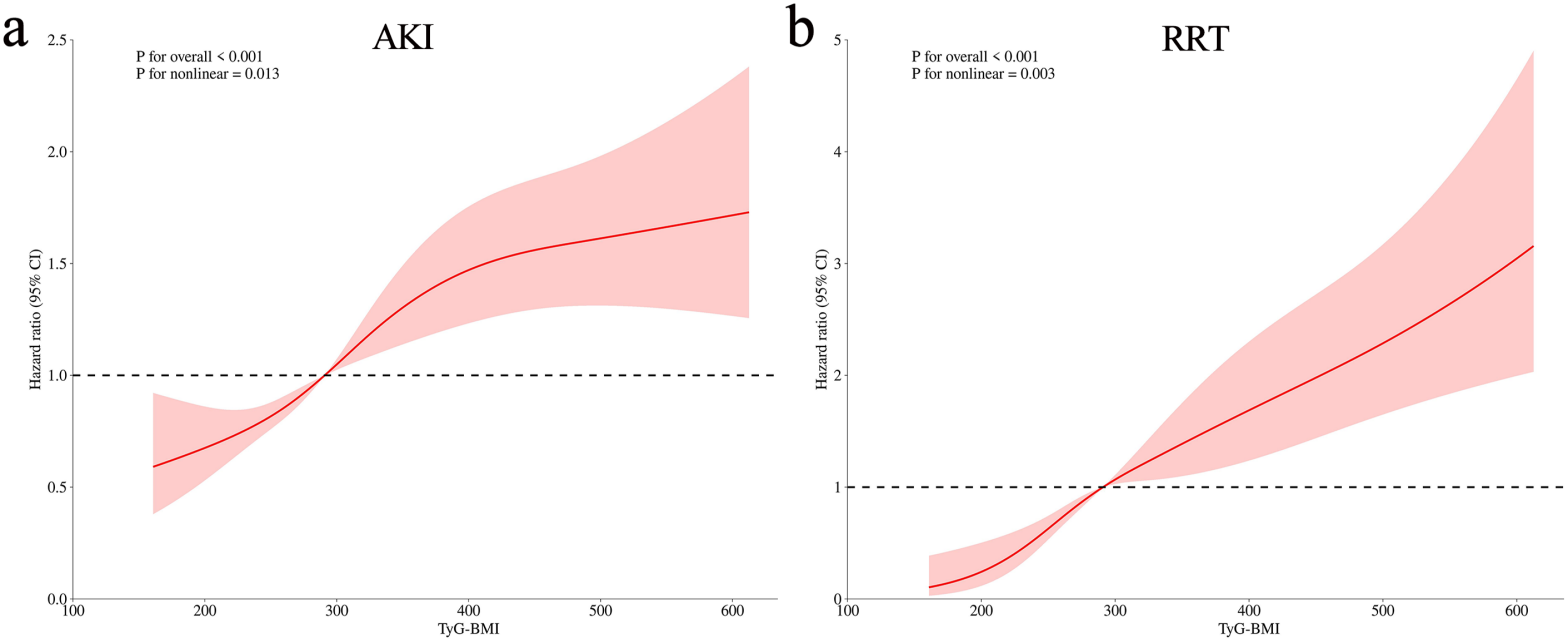
Restricted cubic spline analysis of TyG–BMI index with AKI **(a)** and requiring RRT **(b)**.

The variance inflation factors were calculated to exclude the collinearity of the factors included in the multivariate analysis (**Supplementary Table S1**). The TyG–BMI index was incorporated in Cox regression analysis to identify an independent association with AKI and RRT when, as a continuous variable in model 3, the risk of AKI increased by 1.1% for each 10-unit increase in the TyG–BMI index (*P* = 0.032) (**Table 3**). When incorporated as a nominal variable in model 3, the Q4 group showed a much higher risk of AKI compared to the Q1 group (*P* = 0.012) (**Table 3**); the E-value for this model was 1.73 (**Supplementary Figure S4a**). Similar results were shown for RRT, with a 2.6% increase in AKI risk for a 10-unit increase in the TyG–BMI index (*P* < 0.001) (**Table 3**). In the nominal variable model, significant differences were also observed between groups, with an E-value of 2.05 (**Supplementary Figure S4b**). Meanwhile, the results of the competitive risk analysis using the Fine–Gray model were similar to those of the Cox regression analysis (**Supplementary Table S2**). Furthermore, the RCS analysis demonstrated that the risk of both AKI (*P* for nonlinear = 0.013) and RRT (*P* for nonlinear = 0.003) were nonlinearly associated with increasing TyG–BMI index (**Figure 3**).

**Table 3:** Cox proportional hazard ratios (HR) for AKI and requiring RRT.

Categories	Model 1			Model 2			Model 3		
	HR (95% CI)	*P*-value	*P* for trend	HR (95% CI)	*P*-value	*P* for trend	HR (95% CI)	*P*-value	*P* for trend
AKI incidence									
Continuous variable per 10 units	1.023 (1.015–1.030)	<0.001		1.024 (1.017–1.032)	<0.001		1.011 (1.001–1.021)	0.032	
Quartile			<0.001			<0.001			0.010
Q1 (*N* = 279)	Ref			Ref			Ref		
Q2 (*N* = 279)	1.197 (0.920–1.557)	0.180		1.185 (0.911–1.541)	0.206		1.121 (0.818–1.536)	0.477	
Q3 (*N* = 279)	1.567 (1.216–2.020)	<0.001		1.543 (1.195–1.993)	<0.001		0.998 (0.729–1.367)	0.990	
Q4 (*N* = 280)	2.056 (1.614–2.619)	<0.001		2.145 (1.680–2.738)	<0.001		1.485 (1.092–2.020)	0.012	
Requirement of RRT									
Continuous variable per 10 units	1.045 (1.035–1.056)	<0.001		1.044 (1.034–1.055)	<0.001		1.026 (1.011–1.041)	<0.001	
Quartile			<0.001			<0.001			0.040
Q1 (*N* = 279)	Ref			Ref			Ref		
Q2 (*N* = 279)	2.012 (1.110–3.646)	0.021		1.982 (1.093–3.592)	0.024		1.744 (0.882–3.449)	0.110	
Q3 (*N* = 279)	4.626 (2.681–7.982)	<0.001		4.269 (2.471–7.378)	<0.001		2.147 (1.115–4.132)	0.022	
Q4 (*N* = 280)	5.323 (3.118–9.088)	<0.001		5.044 (2.951–8.621)	<0.001		2.502 (1.312–4.770)	0.005	

Model 1, unadjusted; Model 2, adjusted for age and sex; Model 3, adjusted for age, sex, SOFA, SAPSII, SIRS, platelets, WBC, SCr, BUN, potassium, sodium chloride, ALT, total bilirubin, hemoglobin, RBC, INR, MBP, neutrophils, HF, CHD, AF, diabetes, and cancer.

### Subgroup analyses

**Figure 4:**
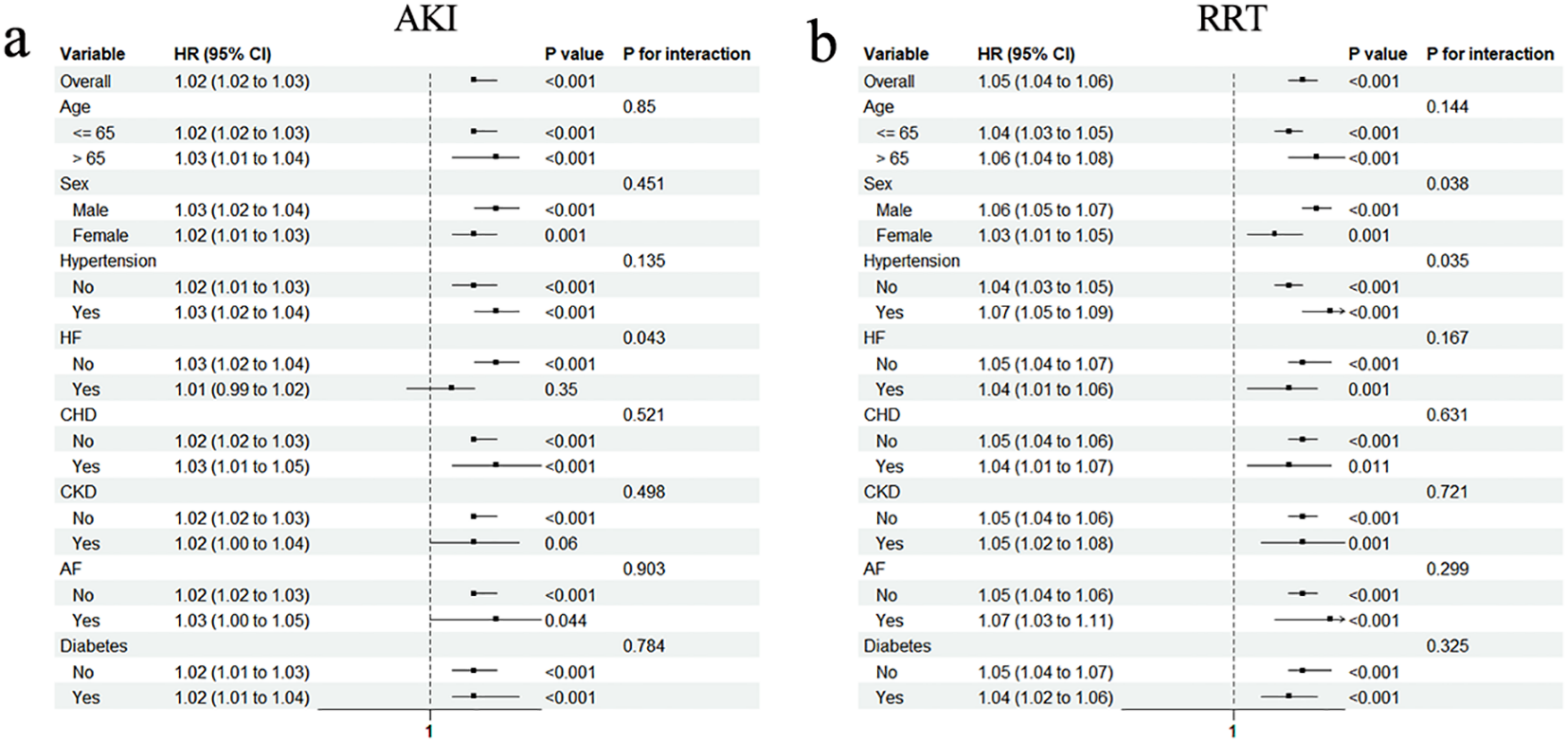
Subgroup analyses for the association of TyG–BMI index with AKI **(a)** and requiring RRT **(b)**. HR, hazard ratio; CI, confidence interval; HF, heart failure; CHD, coronary heart disease; CKD, chronic kidney disease; AF, arterial fibrillation.

To test whether these associations persist in specific populations, subgroup analyses were conducted. The significant association between AKI and the TyG–BMI index persisted in most subgroups, except for patients with HF and CKD (**Figure 4a**). Notably, the association was not as pronounced for patients with HF as it was for patients without HF (HR [95% CI] non-HF: 1.03 [1.02–1.04] vs. HF: 1.01 [0.99–1.02], *P* for interaction = 0.043). However, the prevalence of CKD did not influence the association between AKI and the TyG–BMI index (*P* for interaction = 0.498). Interestingly, all subgroups of the population experienced an increased risk of RRT with higher TyG–BMI index values (all *P* < 0.05; **Figure 4b**).

### Added predictive value of the TyG–BMI index for AKI

To determine the predictive power of severity of illness scores and the combination with TyG–BMI index for sepsis-associated AKI, the AUCs were calculated. The findings indicated a slight increase in ACUs for SOFA, APSIII, SAPSII, and SIRS when the TyG–BMI index was included (**Table 4**). For assessing the risk reclassification power, the NRIs and IDIs were computed next. The results showed that, for severity of illness scores (SOFA, APSIII, SAPSII, and SIRS), the combined use of the TyG–BMI index led to a statistically significant increase in NRI and IDI (all *P* < 0.05) (**Table 4**).

**Table 4:** Performance metrics of severity of illness scores with and without TyG–BMI index to predict sepsis-associated AKI.

	AUC (95% CI)	*P*-value	NRI (95% CI)	*P*-value	IDI (95% CI)	*P*-value
SOFA	0.745 (0.713–0.788)	<0.001				
SOFA + TyG–BMI	0.756 (0.725–0.788)	<0.001	0.010 (0.001–0.022)	0.040	0.011 (0.001–0.020)	0.040
APSIII	0.732 (0.699–0.765)	<0.001				
APSIII + TyG–BMI	0.747 (0.715–0.779)	<0.001	0.007 (0.000–0.016)	0.039	0.006 (0.000–0.017)	<0.001
SAPSII	0.708 (0.674–0.742)	<0.001				
SAPSII + TyG–BMI	0.728 (0.695–0.761)	<0.001	0.028 (0.011–0.049)	<0.001	0.030 (0.009–0.046)	<0.001
SIRS	0.566 (0.531–0.601)	<0.001				
SIRS + TyG–BMI	0.661 (0.625–0.696)	<0.001	0.024 (0.006–0.042)	<0.001	0.019 (0.006–0.036)	0.02

AUC, area under the receiver operating characteristic curve; NRI, net reclassification improvement; IDI, integrated discrimination improvement; SOFA, sequential organ failure assessment; SIRS, systemic inflammatory response syndrome; APSIII, acute physiology score III; SAPSII, simplified acute physiological score II.

## Discussion

The present study is the first to examine the link between the TyG–BMI index and AKI in patients with sepsis. It determined that TyG–BMI exhibited an independent association with AKI, even following adjustment for potential confounding variables, providing a simple and effective predictive tool.

Sepsis often leads to AKI, which not only results in extremely high mortality but also increases the risk of chronic comorbidities (20). Despite ongoing efforts and research, the complex pathophysiology of sepsis-associated AKI has not yet been fully revealed (21). Systemic inflammation and microcirculatory disturbances in the organs were previously thought to be the key mechanisms leading to AKI, but metabolic disturbances during sepsis have attracted increasing attention in recent years (21). During sepsis, elevated catecholamines, release of inflammatory factors, and energy deficits may lead to abnormal lipid metabolism, which, in turn, promotes elevated levels of free fatty acids (22). Meanwhile, tumor necrosis factor can directly inhibit lipoprotein lipase, leading to elevated triglycerides (23). In addition, the release of inflammatory mediators increases liver gluconeogenesis and leads to peripheral IR, resulting in hyperglycemia, even in those without diabetes (24). Hyperglycemia can then trigger ketoacidosis and cause hyponatremia due to high osmolarity, exacerbating kidney damage (25–27). In addition, insulin-like growth factor-binding protein, an important factor involved in IR, has also been found to be directly involved in renal tubule injury (28–30). The abnormalities in blood glucose-related indices have also been shown to be associated with the prognosis of patients with aortic dissection and stroke (31, 32). IR is now recognized as an important causative factor in kidney injury (33).

The TyG–BMI index has been proposed in recent years and proven to better reflect systemic IR in stable populations. Septic patients often experience stress-induced hyperglycemia due to systemic inflammation and catecholamine surge, which may cause changes in the TyG–BMI index to be not only related to IR but also affected by glucose metabolic status (34). This implies that the TyG–BMI index may not accurately represent the state of IR in the body during stress. The specific mechanisms may need to be explored through glucose clamp technique, yet such studies have not been conducted in septic patients so far. Nevertheless, numerous studies have demonstrated its application value in septic patients. Fang et al. demonstrated an independent association between the TyG index and an increased risk of delirium in septic patients (35). In our study, after excluding many confounding factors, the TyG–BMI index was found to be closely related to AKI in the current study; those with a higher index were more prone to AKI. When AKI is progressively aggravated, RRT could address metabolic dysfunction and volume excess, reducing the burden on the kidneys (36). Therefore, the need for RRT is often regarded as an endpoint to represent the progression of AKI severity (37). In the current study, when the TyG–BMI index rose, the incidence of RRT also increased, with a non-linear correlation. Although whether the association between the TyG–BMI index and the study results is attributable to insulin resistance remains unclear, its predictive ability for sepsis-associated AKI and RT is still significant.

To further identify specific populations to which the TyG–BMI index applies, subgroup analyses were performed. The current study showed that the application value of the TyG–BMI index for AKI was not significantly altered by following clinical conditions, including age, sex, hypertension, CHD, AF, and diabetes. However, no significant correlation was found in patients with HF and CKD, which may be partly explained by the fact that HF and CKD are important precipitants to AKI (38, 39). In the context of these diseases, the role of IR and disordered glucose metabolism caused by severe sepsis in the development of AKI may be overshadowed.

The severity of illness scores is a useful tool to objectively quantify disease severity, which helps to identify the disease status and predict its endpoint (40). A previous study has shown that the SOFA score alone did not display a favorable predictive value for major renal adverse events associated with sepsis (41). It is of great value to explore whether combining the severity score with the TyG–BMI index could improve the predictive power. The current results show that the severity of illness scores, when combined with the TyG–BMI index, could significantly improve the ability to predict sepsis-associated AKI. Although it has previously been demonstrated that combining SOFA score with some biomarkers such as calprotectin and cystatin C could also improve the prediction of AKI, these biochemical indices are not routinely tested in most patients, making it easy to miss high-risk patients (42). In contrast, the TyG–BMI index does not increase the financial burden on patients and has the advantages of being simple and easily accessible. The deficiency is that the increment of AUC after combining the TyG–BMI index is relatively small, which seems to lead to a limited clinical impact. However, due to the large number of patients with sepsis and the prevalence of AKI, even a limited increase may bring certain benefits to patients.

### Study strengths and limitations

In the current study, a large cohort was used to confirm the relationship between the TyG–BMI index and sepsis-associated AKI for the first time, and the data were analyzed according to different populations. However, several limitations remain. First, given the retrospective nature of the study, selection bias was unavoidable. Second, some important clinical data, such as infection site, procalcitonin, and C-reactive protein, were not included in the study due to insufficient database information. Third, the current study focused only on assessing the baseline values of the TyG–BMI index, ignoring dynamic changes throughout the treatment period. Fourth, there is currently no direct evidence to establish that the association between the TyG–BMI index and AKI is entirely attributable to IR. Future studies using glucose clamp techniques are warranted to further elucidate this relationship. Finally, since the FBG is inferred rather than explicitly recorded in the database, it may lead to deviations from the true FBG. These deviations may affect the accuracy of the TyG–BMI index and cause discrepancies between subsequent clinical applications and study findings. Therefore, conducting prospective cohort studies in the future is essential.

## Conclusions

The current study demonstrated that the TyG–BMI index is independently associated with AKI and RRT in critically ill patients with sepsis in a nonlinear manner. This suggests that the TyG–BMI index could be a valuable clinical risk classification tool. Strengthening the detection of patients’ TyG–BMI index in clinical practice may help to identify those at a high risk of AKI and enable early intervention to improve the prognosis. Future studies should validate these findings in clinical practice and explore the underlying mechanisms.

## Data Availability

The original contributions presented in the study are included in the article/**Supplementary Material**. Further inquiries can be directed to the corresponding author.

## Supplementary material

Supplementary Figure 1 Visualization of Schoenfeld residuals.

Supplementary Figure 2 Cumulative incidence curves by cumulative incidence function. **(a)** AKI. **(b)** RRT.

Supplementary Figure 3 Kaplan–Meier survival analysis curve. **(a)** ICU. **(b)** In hospital. **(c)** 28 days. **(d)** 1-year mortality.

Supplementary Figure 4 E-value analysis. **(a)** AKI. **(b)** RRT.
