# Supplementary figures and images for: Association between triglyceride glucose–body mass index and acute kidney injury and renal replacement therapy in critically ill patients with sepsis: analysis of the MIMIC-IV database
Source: Front Endocrinol (Lausanne). 2025 Jul 21;16:1561228. doi: 10.3389/fendo.2025.1561228 (PMC12318719; doi:10.3389/fendo.2025.1561228)

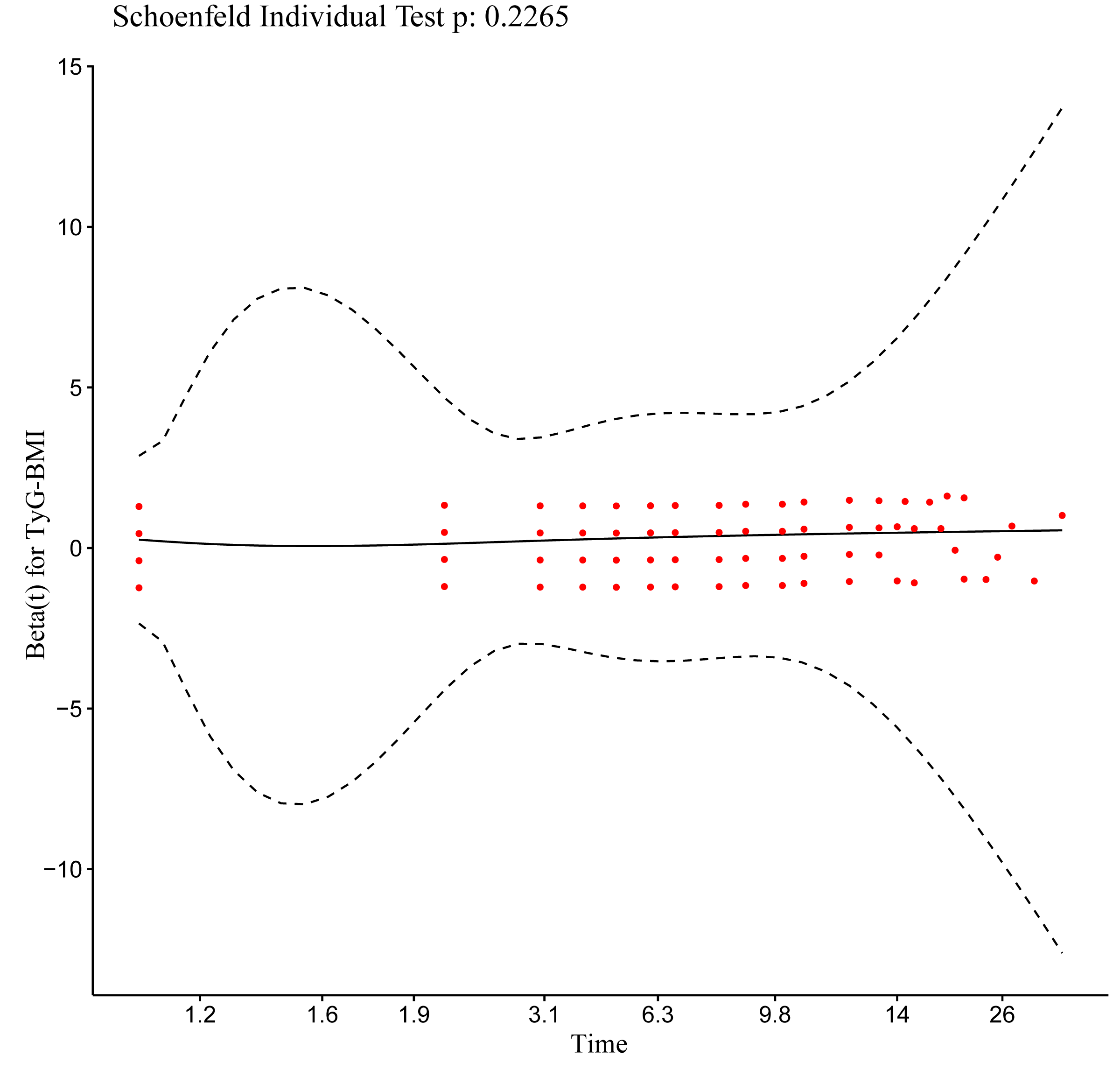

Supplement: Supplementary Figure 1 — Visualization of Schoenfeld residuals. [file Image1.tif]

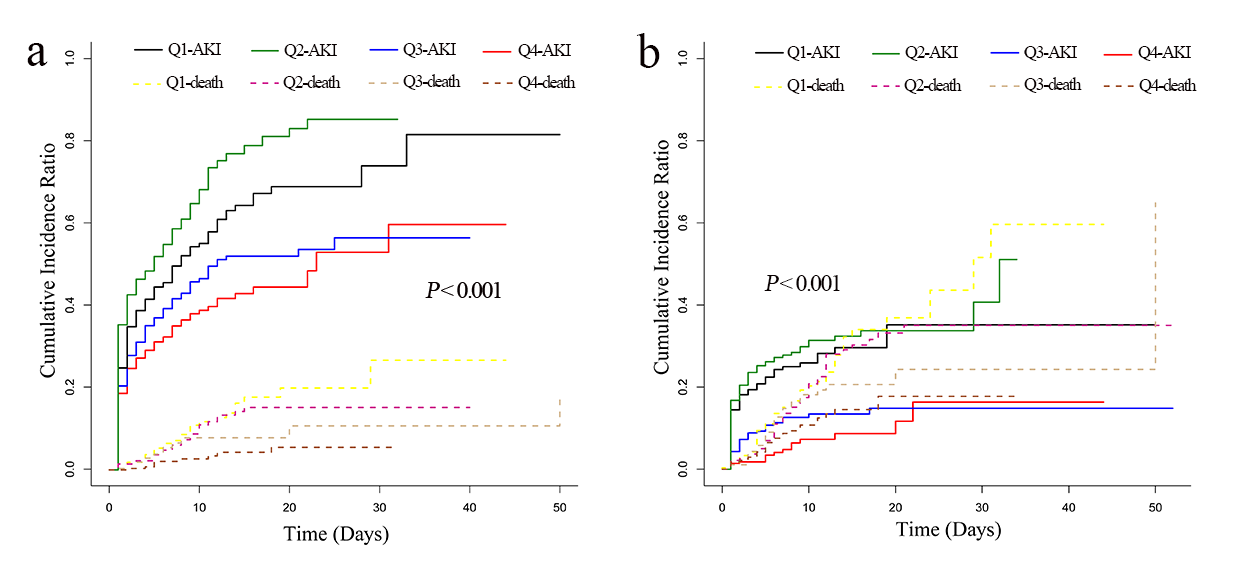

Supplement: Supplementary Figure 2 — Cumulative incidence curves by cumulative incidence function. (a) AKI. (b) RRT. [file Image2.tif]

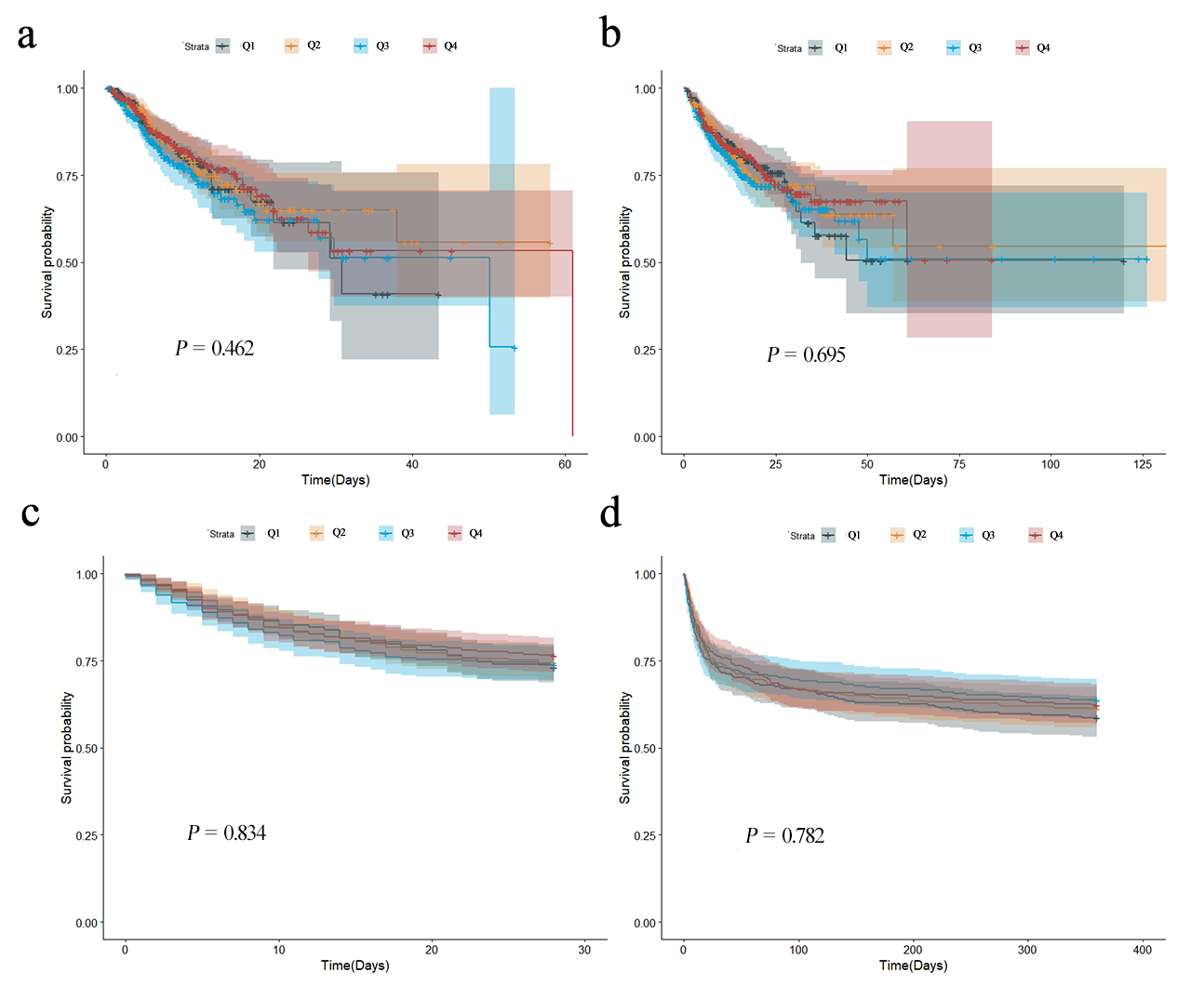

Supplement: Supplementary Figure 3 — Kaplan–Meier survival analysis curve. (a) ICU. (b) In hospital. (c) 28 days. (d) 1-year mortality. [file Image3.tif]

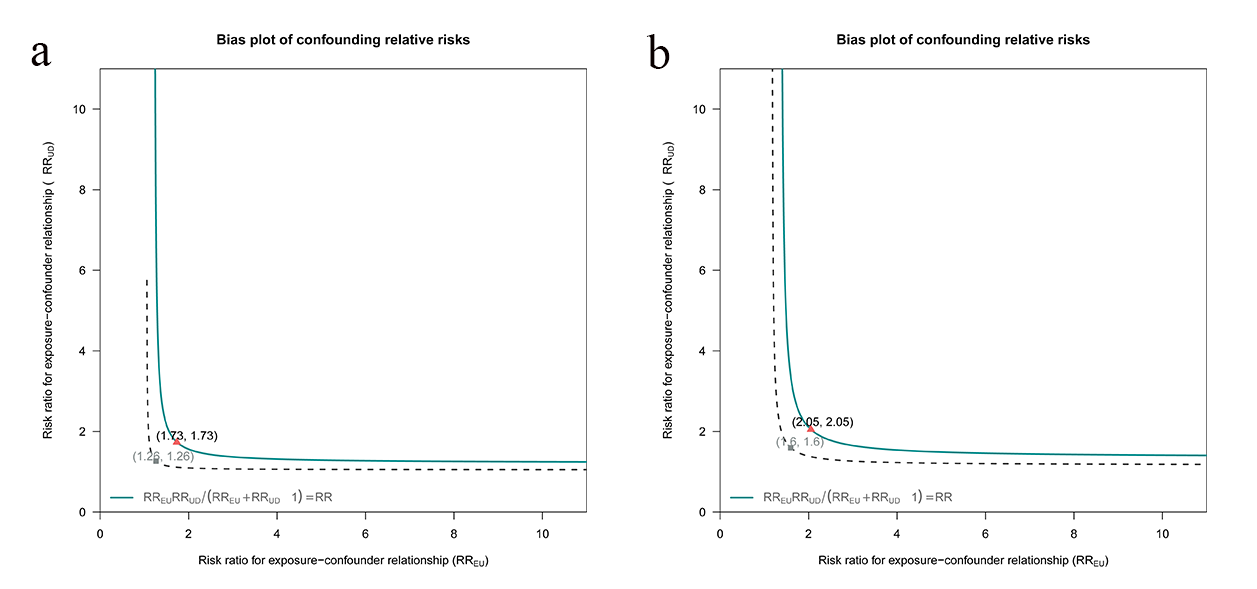

Supplement: Supplementary Figure 4 — E-value analysis. (a) AKI. (b) RRT. [file Image4.tif]
