# Supplementary material for: Association between triglyceride glucose–body mass index and acute kidney injury and renal replacement therapy in critically ill patients with sepsis: analysis of the MIMIC-IV database
Source: Front Endocrinol (Lausanne). 2025 Jul 21;16:1561228. doi: 10.3389/fendo.2025.1561228 (PMC12318719; doi:10.3389/fendo.2025.1561228)
Supplement: Supplementary file 5 [file DataSheet1.docx]

**The logic and related** **codes for inferring fasting blood glucose**

**Inference logic:**

The data of blood glucose, insulin, glucose injection and enteral nutrition were extracted by PostgresSQL and Navicat Premium software, and merge with Stata software.

By extracting the blood collection time for glucose tests and comparing it with the start time of insulin, glucose injection, and enteral nutrition. If the blood collection occurred after the start of any of these interventions, the corresponding glucose value was considered interfered and excluded. Conversely, glucose values obtained before the start of these treatments were inferred as FBG.

**Codes:**

1. Extract data from the database
2. Blood glucose

SELECT * FROM d_labitems where label ~* 'Glucose'

SELECT subject_id, hadm_id, charttime, valuenum,valueuom FROM labevents where itemid in ('50809','50931','52569','52027')

1. Insulin

SELECT * FROM d_items where label ~* 'insulin'

SELECT subject_id, subject_id,stay_id, starttime, itemid FROM inputevents where itemid in ('223257','223258','223259','223260','223261','223262','226222','228236','229299','229619')

1. Glucose infusion

SELECT * FROM d_items where label ~* 'Dextrose'

SELECT subject_id, subject_id,stay_id, starttime, itemid FROM inputevents where itemid in ('220949','220950','220951','220952','220963','220964','220965','220966','220967','220968','221000','221002','221014','221017','228140','228141','228142')

1. Enteral nutrition

SELECT * FROM inputevents where ordercategoryname ~* '13-Enteral Nutrition'

SELECT * FROM d_items where itemid in ('225930', '228135','225934','225936','229295','229013','227975','226880','225931','225970','229010','228351','229014','229012','229296','229297','227979','229011','228383','228363','229009','228367','225935','227695','226039','221036','228359','226051','227698','226019','225928','225937','225929','226050','226016','226877','221207','226882','226047','226036','227973','226048','226059','226030','226031','226046','228133','226881','228132','226017','226044','227518','227974','228348','228360','226027','226058','227696','227699','226045','227977','226023','228131','226049','226022','228356','228355','226875','228364','226028','226038','228134','227978','226026','226024','227976','226020','228361')

1. Data merging and screening
2. Blood glucose

intime: Time of admission to the ICU; glucosecharttime: Blood collection time

merge 1:m hadm_id using "Glucose.dta"

gen intime2 = substr( intime ,1,13)

gen intimehour = clock( intime2 ,"YMDh") /3600/1000

gen glucosecharttime2 = substr( glucosecharttime ,1,13)

gen glucosehour = clock( glucosecharttime2 ,"YMDh") /3600/1000

drop if glucosehour - intimehour > 24

drop if glucosehour - intimehour < 0

1. Insulin

insulinstarttime: time of starting insulin use; glucosecharttime: Blood collection time

merge m:m stay_id using "Insulin.dta"

gen insulinstarttime2 = substr( insulinstarttime ,1,13)

gen Insulinhour = clock( insulinstarttime2 ,"YMDh") /3600/1000

drop if Insulinhour < glucosehour

1. glucose infusion

Glcstarttime: time of starting glucose infusion

merge 1:m stay_id using "Insulin.dta"

gen Glcstarttime2 = substr( Glcstarttime ,1,13)

gen Glchour = clock( Glcstarttime2 ,"YMDh") /3600/1000

drop if Glchour < glucosehour

1. Enteral nutrition

ENstarttime: the time to start using enteral nutrition

merge 1:m stay_id using " enteral nutrition.dta"

gen ENstarttime2 = substr( ENstarttime ,1,13)

gen ENhour = clock(ENstarttime2 ,"YMDh") /3600/1000

drop if ENhour < glucosehour
