# Supplementary material for: Association between triglyceride glucose–body mass index and acute kidney injury and renal replacement therapy in critically ill patients with sepsis: analysis of the MIMIC-IV database
Source: Front Endocrinol (Lausanne). 2025 Jul 21;16:1561228. doi: 10.3389/fendo.2025.1561228 (PMC12318719; doi:10.3389/fendo.2025.1561228)
Supplement: Supplementary file 6 [file Table1.docx]

| **Variables** | **VIFs** |
| --- | --- |
| Age | 1.751 |
| Sex | 1.180 |
| SOFA | 3.567 |
| SAPSII | 3.182 |
| SIRS | 1.283 |
| Platelets | 1.273 |
| WBC | 1.108 |
| SCr | 3.681 |
| BUN | 1.487 |
| Potassium | 1.255 |
| Sodium | 2.194 |
| Chloride | 2.252 |
| ALT | 1.185 |
| Total bilirubin | 1.493 |
| Hemoglobin | 3.457 |
| RBC | 3.248 |
| INR | 3.757 |
| MBP | 1.315 |
| Neutrophils | 1.437 |
| HF | 1.183 |
| CHD | 1.256 |
| AF | 1.099 |
| Diabetes | 1.181 |
| Cancer | 1.120 |
| TyG-BMI | 1.327 |

**Supplementary Table 1:** The variance inflation factors of the factors included in the multivariate analysis.

VIFs: variance inflation factors; SOFA, sequential organ failure assessment; SOFA, sequential organ failure assessment; SIRS, systemic inflammatory response syndrome; SAPSII, simplified acute physiological score II; SIRS, systemic inflammatory response syndrome; WBC, white blood cell; SCr, serum creatinine; BUN, blood urea nitrogen; ALP, alkaline phosphatase; RBC, red blood cell; INR, international normalized ratio; MBP, mean blood pressure; HR, heart rate; CHD, coronary heart disease; AF: Arterial fibrillation; TyG-BMI: triglyceride glucose-body mass index
