# Supplementary material for: Association between triglyceride glucose–body mass index and acute kidney injury and renal replacement therapy in critically ill patients with sepsis: analysis of the MIMIC-IV database
Source: Front Endocrinol (Lausanne). 2025 Jul 21;16:1561228. doi: 10.3389/fendo.2025.1561228 (PMC12318719; doi:10.3389/fendo.2025.1561228)
Supplement: Supplementary file 7 [file Table2.docx]

| Categories | Model1 |  | Model2 |  | Model3 |  |
| --- | --- | --- | --- | --- | --- | --- |
|  | HR (95% CI) | *P*-value | HR (95% CI) | *P*-value | HR (95% CI) | *P*-value |
| AKI incidence |  |  |  |  |  |  |
| Quartile |  |  |  |  |  |  |
| Q1(N=279) | Ref | <0.001 | Ref | <0.001 | Ref | 0.0425 |
| Q2(N=279) | 1.202(0.932-1.550) | 0.156 | 1.188(0.924-1.529) |  | 1.117(0.881-1.416) | 0.3609 |
| Q3(N=279) | 1.604(1.261-2.040) | <0.001 | 1.582(1.247-2.008) |  | 1.125(0.890-1.422) | 0.3245 |
| Q4(N=280) | 2.113(1.690-2.612) | <0.001 | 2.196(1.749-2.757) |  | 1.527(1.214-1.921) | 0.0003 |
| Requirement of RRT |  |  |  |  |  |  |
| Quartile |  |  |  |  |  |  |
| Q1(N=279) | Ref | <0.001 | Ref | <0.001 | Ref | 0.0031 |
| Q2(N=279) | 1.982(1.103-3.562) |  | 1.953(1.085-3.515) |  | 1.587(0.888-2.837) |  |
| Q3(N=279) | 4.711(2.783-7.974) |  | 4.356(2.570-7.381) |  | 2.272(1.330-3.882) |  |
| Q4(N=280) | 5.301(3.143-8.941) |  | 5.031(2.973-8.514) |  | 2.578(1.502-4.425) |  |

Supplementary Table 2. Cox proportional hazard ratios (HR) for AKI and requiring RRT

Model 1: unadjusted

Model 2: adjusted for age, sex

Model 3: adjusted for age, sex, SOFA, SAPSII, SIRS, platelets, WBC, SCr, BUN, potassium, sodium, chloride, ALT, total bilirubin, hemoglobin, RBC, INR, MBP, neutrophils, HF, CHD, AF, diabetes, cancer
